# QRFXFreeze: Queryable Compressor for RFX

**DOI:** 10.1155/2015/864750

**Published:** 2015-05-06

**Authors:** Radha Senthilkumar, Gomathi Nandagopal, Daphne Ronald

**Affiliations:** ^1^Department of Information Technology, Anna University, MIT Campus, Chennai, Tamil Nadu 600044, India; ^2^Computer Science and Engineering, Vel Tech University, Avadi, Chennai, Tamil Nadu 600062, India

## Abstract

The verbose nature of XML has been mulled over again and again and many compression techniques for XML data have been excogitated over the years. Some of the techniques incorporate support for querying the XML database in its compressed format while others have to be decompressed before they can be queried. XML compression in which querying is directly supported instantaneously with no compromise over time is forced to compromise over space. In this paper, we propose the compressor, QRFXFreeze, which not only reduces the space of storage but also supports efficient querying. The compressor does this without decompressing the compressed XML file. The compressor supports all kinds of XML documents along with insert, update, and delete operations. The forte of QRFXFreeze is that the textual data are semantically compressed and are indexed to reduce the querying time. Experimental results show that the proposed compressor performs much better than other well-known compressors.

## 1. Introduction

XML is becoming increasingly popular in the developer community as a tool for passing, manipulating, storing, and organizing information [1, 2]. In real time XML documents are generally huge in size. The markup in the XML document (attributes, tags, etc.) contributes a significant amount to overall size of it. This is attributed to the large size of XML documents. Nevertheless, the advantages of storing information in XML format had placed an ever increasing demand for high performance XML storage and querying systems. The proposed work focuses on designing a compressor for XML documents which would also support fast querying. The conventional method of querying an XML system is to parse the document and create an in-memory representation of the document. The in-memory representation is usually many times the size of the original XML file which sometimes results in out of memory errors. So there is a need for an efficient storage mechanism to minimize the in-memory representation for the XML documents which also supports fast querying along with other navigational operations such as insert, update, and delete. The previous work, RFX (Redundancy Free XML storage structure) [3, 4], is one such storage scheme that results in dramatic improvements in memory usage and scalability and significant improvements in performance. The key advantage of this scheme is that the XML document is not stored in the form of a tree which eliminates the need for pointers. Also, separate the structure of the XML documents from its textual content so as to treat the textual data separately while compressing. The QRFXFreeze proceeds further to compress and index the data stored in RFX storage structure and design a querying method that retrieves the data from the compressed structure without decompressing the compressed storage structure. The generic nature of the XML document does not allow indexing since the values are not stored according to any key values. In QRFXFreeze, the data is indexed to achieve query efficiency. Thus the optimal balance between storage space and querying is achieved.

## 2. Related Work

XML compression techniques [5] examine XML compressors in various experimental setups using XML documents of varying size and nature. The works on nonqueryable compressors followed by queryable one are discussed. XMill [6] is one of the oldest compressors developed for XML databases and remains one of the most efficient nonqueryable compressors even after almost a decade. The idea of splitting structure and text into containers gives rise to the possibility of semantic compressors and this hugely increases the compression ratio. However, the major disadvantage of XMill is that it is nonqueryable and hence the querying time increases substantially. The Millau [7, 8] coding format is an extension of WAP (Wireless Application Protocol) Binary XML (WBXML) format. However, it does not reorganize its data and hence suffers a lower compression ratio than others. Cheney proposed XMLPPM [9, 10] in which several hierarchical models are used and the major disadvantage is that the compression time is relatively longer than other compressors. XGrind [11] was the first XML compressor that supported queries in the compressed domain. However, the variety of queries supported is limited and newer tools have overtaken XGrind by processing queries with minimal querying time. XPress [12] is similar to XGrind and adopts a homomorphic transformation strategy to transform an XML document into a compressed form that preserves the syntactic and semantic information of the original XML document. It performs querying in lesser time than XGrind. In [9], Cheney proposes XMLPPM, a streaming XML compressor, where the XML file is first parsed by an SAX parser. The generated bytecodes are encoded again in the PPM model based on the structure of the XML document. SCMPPM [13] is a variant of the XMLPPM compressor applies the text compression to the content of each element. Hence it uses larger set of PPM models than XMLPPM and combines Structure Context Modeling (SCM) with the PPM compression technique. Exalt [14] uses grammar transform operation reported in the work of Kieffer and Yang to produce irreducible grammar. This is then encoded with an adaptive arithmetic coder to compress the XML document. AXECHOP [15] treats the structural and data part of the document differently and encodes the data with BWT algorithm. It compresses the structure with MPM. RFXFreeze [16], a nonqueryable compressor for RFX storage structure, attains a high compression ratio at the cost of time for efficient retrieval of data. XCQ [17] uses DTD information to facilitate efficient querying. One problem may be that all XML databases do not have DTDs. XQueC [18] is a compressed XML database rather than a mere compression tool. It supports querying without compromising on the compression efficiency. But it leaves the update problem unexplored. XQzip [19] uses a queryable storage model for compressed data. It employs indexing scheme to improve query performance in the compressed format. XWRT (XML Word Replacing Transform) [20] uses a transformation called XWRT and a reverse XWRT to perform compression. It uses a dictionary-based compression technique to eliminate redundancy which in turn leads to good results. DataXSeq [21] takes a similar approach but compresses both data and structure separately with Sequitur. An interesting feature is its possibility of processing queries directly over the compressed file. XSeq follows sequence based XML indexing and grammar based text string compression algorithm. XCpaqs [22] also separates the structure and context. It achieves good compression ratio and fast query processing. Though it supports long XPATH queries, complex operators such as aggregation and join are unexplored. ISX [23], a new compact XML storage engine, to store XML in a more concise structure. Theoretically, ISX uses an amount of space near the information theoretic minimum on random trees. But it is a schema aware [24] storage system.

## 3. Modification to RFX Structure

Redundancy Free XML storage structure (RFX) is a multilayered architecture where the element and data are stored separate layers and this facilitates the navigation and retrieval of data easily. RFX has been implemented using a succinct storage representation where the relationship between XML tags and attributes is represented using bits. This reduces the storage size by achieving high compression ratio. Thus RFX storage scheme achieves optimal balance between the storage and query efficiency. In QRFXFreeze, the structure of RFX [21] is altered in order to make the querying more efficient. The main modification is introducing containers in the data layer. The following explains the concept of these containers.

### 3.1. Concept of Containers

**Figure 1:**
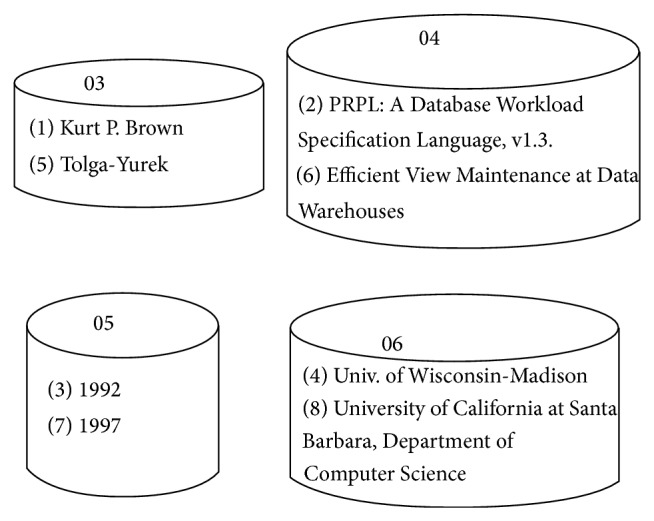
Snippet of container for DBLP.XML.

The Data Layer of the RFX has been modified by using containers instead of “Element Data Table” and “Attribute Data Table.” The elements or attributes with the same Element ID or Attribute ID, respectively, are taken together and put in the same container. So for each element tag in the XML, there would be a data container containing the Element Data values for a particular element. For example, consider the Element Data Table in RFX for a fragment of dblp.xml given in Table 1. Applying the concept of containers, the Element Data Table in Table 1 would be transformed into the containers as shown in Figure 1.

*Benefit of Containers.* The complete set queries or unconditional queries can be executed with ease in a less amount of time. Each container is compressed contextually; that is, different types of compression can be applied based on whether the data is text or alphanumeric or integers and so forth. Since each container is identified using the Element ID, the access of the container is easier and cost-efficient. Obviously, searching for a particular data item becomes much faster because the size of a container is 1/*e* size of the Element Data Table, where *e* is the number of elements in the element table.

## 4. Proposed Architecture

**Figure 2:**
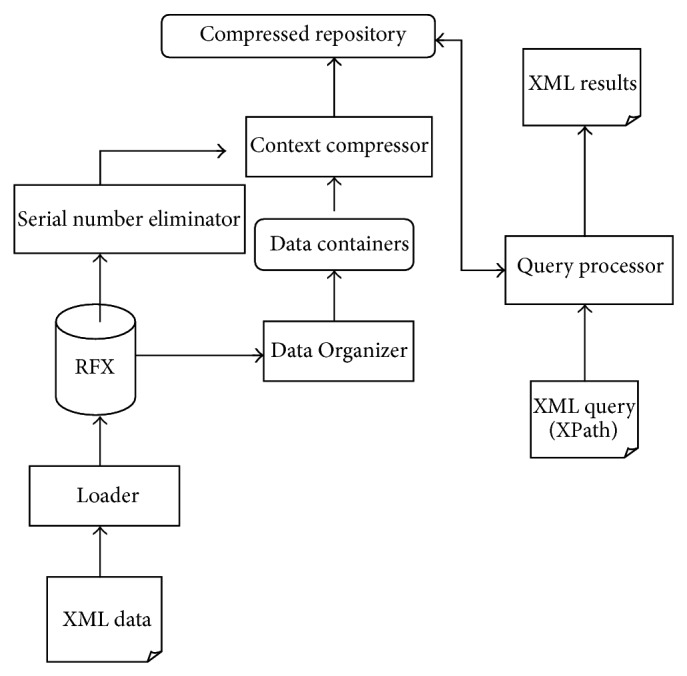
Architecture of QRFXFreeze.

The architecture of QRFXFreeze shown in the Figure 2 mainly consists of the following.

**Figure 3:**
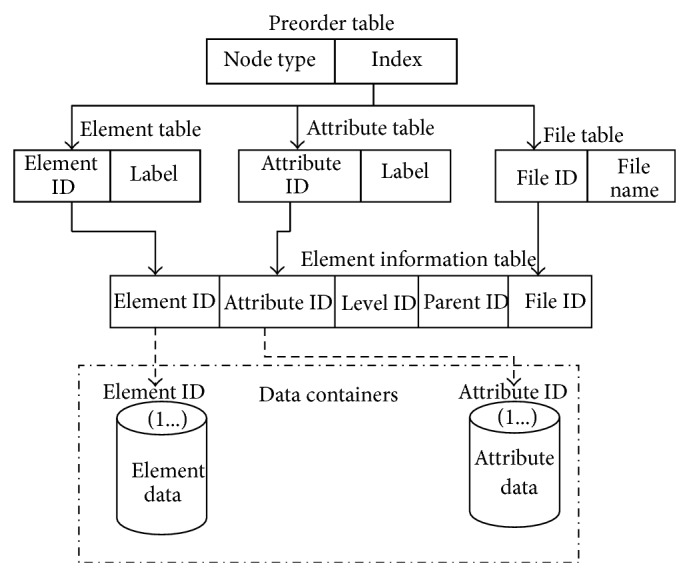
QRFXFreeze storage structure.

- QRFXFreeze storage manager: RFX database is given as the input and the equivalent QRFXFreeze database is obtained as output. The structure of a QRFXFreeze database is depicted in Figure 3.
- QRFXFreeze query processor: Query is taken as a input, processes it and returns the results.

The Loader takes as input an XML document and parses it and stores it in the form of RFX. The Element ID and Attribute IDs in the Element and Attribute tables of the layer 2 in the RFX structure are in serial order and hence can be removed by Serial Number eliminator. The Data Organizer is the component that splits the data into containers. The data which have the same Element ID or Attribute ID, whichever applies, are consolidated into the same container.

The Loader takes as input an XML document and parses it and stores it in the form of RFX. The Element ID and Attribute IDs in the Element and Attribute tables of the layer 2 in the RFX structure are in serial order and hence can be removed by Serial Number eliminator. The Data Organizer is the component that splits the data into containers. The data which have the same Element ID or Attribute ID, whichever applies, are consolidated into the same container. The algorithm for the Data Organizer is as given in Algorithm 1.

The text compressor [25] builds a string translation table from the text being compressed. The string translation table maps fixed-length codes (usually 12-bit) to strings. The string table is initialized with all single-character strings (256 entries in the case of 8-bit characters). As the compressor character serially examines the text, it stores every unique two-character string into the table as a code/character concatenation with the code mapping to the corresponding first character. As each two-character string is stored, the first character is sent to the output. Whenever a previously encountered string is read from the input, the longest such previously encountered string is determined, and then the code for this string concatenated with the extension character (the next character in the input) is stored in the table. The code for this longest previously encountered string is output and the extension character is used as the beginning of the next word. The text compression algorithm is depicted in Algorithm 2.

The modified structure is now passed onto the query processor which takes any input query, does the required processing, and returns the output to the user.

## 5. QRFXFreeze Query Processor

### 5.1. Query Processor

A query processor extracts the high level abstraction of declarative query and its procedural evaluation into a set of low-level operations [26]. For processing a query, the QRFXFreeze processor translates the syntax (parsing and analysis) and then executes the operations expressed by the query. The query output is returned after this processing and the querying time is projected to be minimum, thus adverting efficient processing.

### 5.2. Architecture of QRFXFreeze Query Processor

**Figure 4:**
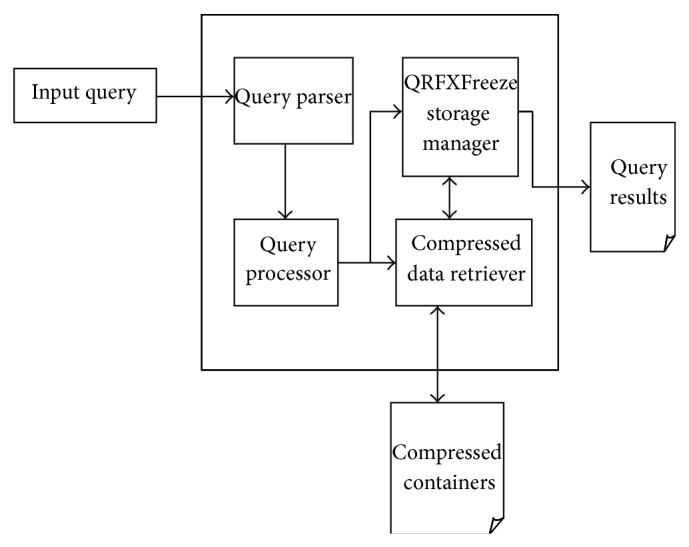
Architecture of QRFXFreeze query processor.

The main components in the query processor as depicted in Figure 4 are the querying component and the storage back-end. The querying component takes care of analyzing the query and validating it and also directs the storage manager for the retrieval of data. The storage back-end contains all the data in compressed format. Its main job is to retrieve and transfer the required data from compressed form and display it as output to the user. The query parser accepts the input query. It then enters an analysis phase. The first step is to validate the query. Once the query is certified to be a valid one, the query parser proceeds to classify whether the input query is a simple, conditional, or a nested query. The query processor takes input from the query parser. The query processor now has information about the type of query that it has to process. The query processor handles each query based on its type. After processing the query, the processor needs access to the actual data. It contacts the storage manager to locate and retrieve the particular data items. The storage manager is directed by the query processor to locate the data. It contains the algorithms for compressing, decompressing data, and also retrieving the data directly without decompressing the entire container. It is responsible for consolidating the results and displaying the output to the user. The input to the query processor is an XPath query which concords to the following grammar provided in [27].

The compressed data retriever of the storage manager facilitates access of compressed data. Text decompression is required to access individual data items in the containers. After the particular container (in case of simple query) or containers (in the case of conditional or correlated queries) are identified, they are decompressed using the text decompression algorithm depicted in Algorithm 3.

### 5.3. Support for Querying

All the 3 basic types of querying can be supported in the QRFXFreeze architecture:

- complete set query or unconditional query;
- conditional query;
- correlated or nested query.

#### 5.3.1. Complete Set Query or Unconditional Query

Complete set queries are the simplest type of queries that the query processor has to handle. It involves simply retrieving the data of an entire leaf or nonleaf node element of the XML tree.

For example, consider the following query for Shakespeare.xml. /PLAYS/PLAY/TITLE.

The above expression can be interpreted as follows: starting from the root of_the XML document (which is represented by ＇/＇) traverse until the 〈PLAYS〉 element is found, then deep traverse to find the 〈PLAY〉 element, and then retrieve the value of the 〈TITLE〉 element. The algorithm for how the QRFXFreeze query processor handles complete set queries is given in Algorithm 4.

#### 5.3.2. Conditional Query

Conditional queries are those in which only the set of data must be displayed which satisfy a predicate given in the query. The condition may be to print details of only a particular element or within a certain range or may contain Boolean operators such as “and,” “or,” and “not equal to”.

Consider the following conditional query for Shakespeare.xml:

 /PLAYS/PLAY/ACT/SCENE [SPEAKER ~ == ~ PHILO].

The query is interpreted as printing all details for the element SCENE in which SPEAKER is PHILO. First, the Element ID of the element SPEAKER is found from the Element Table. Next, the data container with name Element ID is located and the Element Data ID for PHILO is found. Then, using the Order Encoding and then Element Structure mapping files, the Element IDs and Data IDs of all the enclosing tags and data are stored in a buffer. Then the Element IDs are used to find the data containers and the Data IDs are used to locate the actual data. The contents of the buffer are outputted. The algorithm for how query processor handles the conditional query is depicted in Algorithm 5.

#### 5.3.3. Correlated Query

The QRFXFreeze query processor supports correlated or nested queries because the RFX storage structure supports both intra- and inter-XML documents. The query processing algorithm for nested queries adopts the strategy list method proposed in [3]. The algorithm for nested query is given in Algorithm 6.

Consider the following example for nested query:

 //students/student [id = /exam [grade < ‘B']/id]/name “/exam [grade < ‘B']/id” is the repeating subquery. This query involves two different scopes, namely, “students” and “exam.”

## 6. Experimental Results

The six data sources that cover a wide range of XML data formats and structures have been used for the experiments. The test queries are run on various standard benchmarks. The benchmark and its characteristics are given in Table 2.

### 6.1. Performance Analysis of QRFXFreeze

#### 6.1.1. Evaluation Methodology for QRFXFreeze

*Compression Ratio.* We express the compression ratio as the ratio of the size of the compressed document to the original document. For example, if a 10 MB file can be compressed to 2.5 MB, the file is 75% compressed. Higher compression ratios are, obviously, better:

(1) Compression  Ratio% =1−size  of Compressed  filesize  of original  file∗100.

*Querying Time.* Querying time is the time elapsed between the periods when the user enters the query and until the query results are displayed to the user. It includes the query analysis time and query processing time. The lesser the querying time is, the more efficient the queryable compressor is.

**Figure 5:**
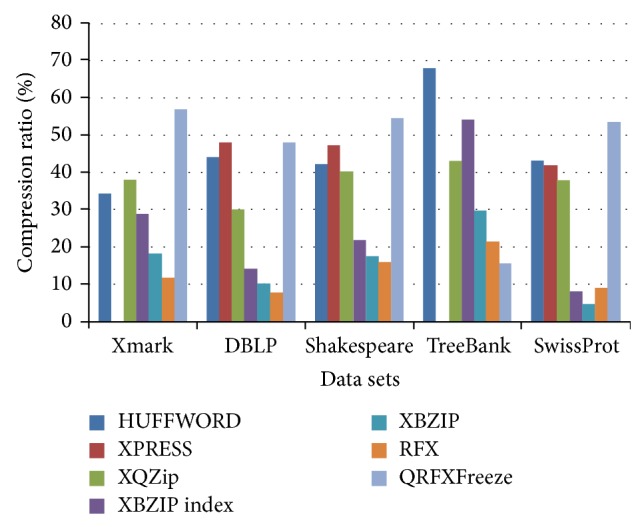
Storage size comparison (compression ratio) of QRFXFreeze with other queryable compressors.

The compression ratios achieved by these compressors are taken from [28]. The comparison ratio of QRFXFreeze with other queryable compressors is depicted in Figure 5. The result of XPRESS compressor for TreeBank and Xmark is not available in [28]. It is observed that the QRFXFreeze outperforms Xmark, DBLP, Shakespeare, and SwissProt. But the same fails for TreeBank dataset as there are especially fewer redundancies in TreeBank dataset.

#### 6.1.2. Querying Time Comparison of QRFXFreeze with Other Queryable Compressors

The following lists of queries for each dataset have been used in the performance evaluation. The query execution times of XQZip and XGrind were taken from [19].

Shakespeare.xml:

Q1. * //PLAY/ACT/SCENE/SPEECH/SPEAKER.*
Q2. * //PLAY/ACT/SCENE/SPEECH[SPEAKER =*  ＇＇*PHILO*＇＇*].*
Q3. * //PLAY/ACT/SCENE/SPEECH[SPEAKER >=*  ＇＇*MARK ANTONY*＇＇* and SPEAKER <=*  ＇＇*PHILO*＇＇*].*

lineitem.xml:

Q4. * /table/T/L_TAX.*
Q5. * /table/T[L_TAX = 0.02].*
Q6. * /table/T[L_TAX >= 0.02 and L_TAX <= 0.04].*

dblp.xml:

Q7. * /dblp/inproceedings/booktitle.*
Q8. * /dblp/inproceedings[booktitle =*  ＇＇*SIGMOD Conference*＇＇*].*
Q9. * /dblp/inproceedings[year >= 1998 and year <= 2000].*

treebank_e.xml:

Q10: * //PP//PP//PP//PP//PP//PP//PP//PP.*
Q11: * //PP[//PP]//NP.*

The graph in Table 3 illustrates the query performance of QRFXFreeze for each data set. The execution times are lesser when compared to other querying systems like XQZip and RFX (the symbol “–” indicates that the result is not available in the paper) [19].

*Nested Queries.* To the best of our knowledge none of the compressors support nested queries which query more than one document. The following were taken as test queries.  Table 4 illustrates the query performance for nested queries.

Files are student.xml and exam.xml.

Q1. /students/student[roll_no~ == ~/exams/exam[course_no~ == ~ CS501]/roll_no]/name:
 Find the names of students who attended exam with course_no CS501.
Q2. /students/student[roll_no ~ == ~ /exams/exam/roll_no]:
 Find students who attended atleast one exam.
Q3. /exams/exam[course_no~ == ~ students/student/course/course_no]/course_name:
 Find the courses for which exam has been conducted.

## 7. Conclusion

The queryable compressor for RFX has been proposed. The experimental results show that QRFXFreeze beats the RFX and other popular XML queryable compressors at both the consumption of storage space and also the querying time. Also the variety of queries supported by the QRFXFreeze when compared to legacy compressors is an added advantage. Furthermore, since the textual data has been separated from the structure, indexing schemes can be applied along with text compression algorithms to facilitate faster access to the data in its compressed format.

## Figures and Tables

**Algorithm 1:**
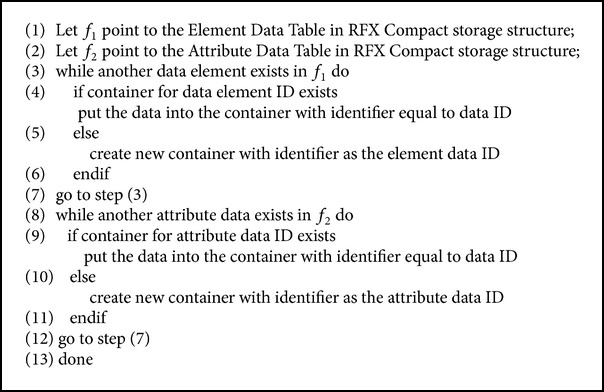
Algorithm for the Data Organizer.

**Algorithm 2:**
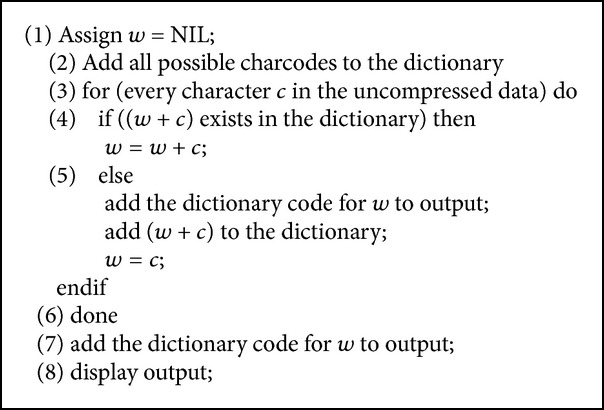
Text compression algorithm.

**Algorithm 3:**
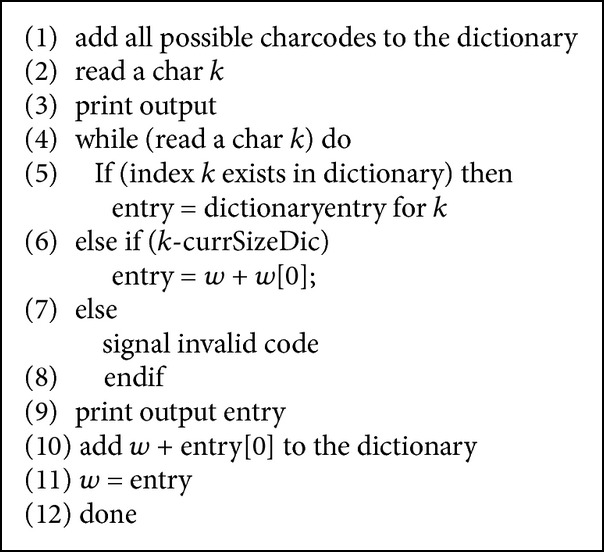
Text decompression algorithm.

**Algorithm 4:**
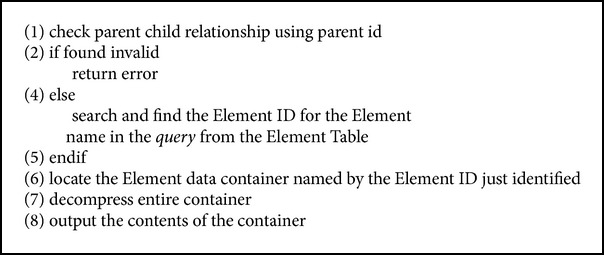
Complete set query.

**Algorithm 5:**
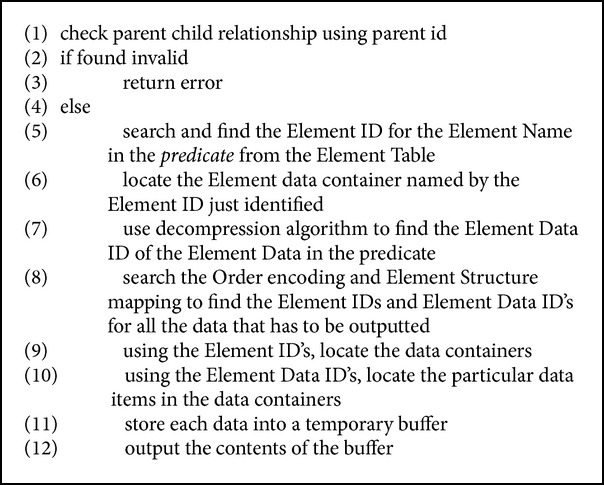
Conditional query.

**Algorithm 6:**
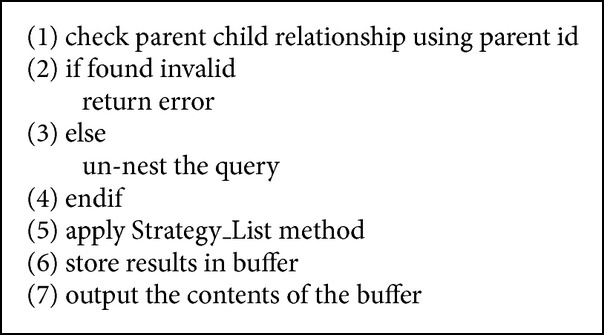
Nested query.

**Table 1:** Snippet of Element Data Table for DBLP.XML.

Element ID	Data ID	Data
03	1	Kurt P. Brown
04	2	PRPL: A Database Workload Specification Language, v1.3.
05	3	1992
06	4	Univ. of Wisconsin-Madison
03	5	Tolga-Yurek
04	6	Efficient View Maintenance at Data Warehouses
05	7	1997
06	8	University of California at Santa Barbara, Department of Computer Science

**Table 2:** Benchmark datasets and their characteristics.

Data source	Size (MB)	Depth	Tags/Attrs.	Nodes
XMark	111	11	86	2018493
DBLP	148	6	41	8594355
TreeBank	82	36	252	2437667
Shakespeare	7.3	6	23	179072
SwissProt	109	5	49	21634330

**Table 3:** Query execution time in seconds on various queryable compressors.

Queries	XQZip	XGRIND	RFX	QRFXFreeze
Q1	0.014	1.311	0.00167	0.0012064
Q2	0.016	1.62	0.000301	0.0001672
Q3	0.016	2.32	0.001128	0.0002573
Q4	0.011	2.336	0.0028	0.0012493
Q5	0.012	2.89	0.00267	0.0006154
Q6	0.014	3.21	0.004074	0.0015997
Q7	0.034	19.582	0.007548	0.0045959
Q8	0.029	26.108	0.001019	0.0002202
Q9	1.543	50.344	0.00601	0.00296825
Q10	0.177	—	0.001817	0.0028135
Q11	0.985	—	0.013875	0.0104238

**Table 4:** Query execution times for nested queries.

Queries	RFX	QRFXFreeze
Q1	0.006425	0.01
Q2	0.017838	0.01679
Q3	0.015743	0.00276
